# Socioeconomic inequalities in treatment of individuals with common mental disorders regarding subsequent development of mental illness

**DOI:** 10.1007/s00127-017-1389-6

**Published:** 2017-05-11

**Authors:** Thomas E. Dorner, Ellenor Mittendorfer-Rutz

**Affiliations:** 1 Centre for Public Health, Department for Social and Preventive Medicine, Medical University of Vienna, Vienna, Austria; 2 Division of Insurance Medicine, Department of Clinical Neuroscience, Karolinska Institutet, Stockholm, Sweden

**Keywords:** Socio-economic status, Education, Suicide, Depression, Anxiety, Stress-related disorders

## Abstract

**Purpose:**

Socioeconomic differences appear to be reflected in both, the development and the treatment of common mental disorders (CMDs, i.e. depressive, anxiety and stress-related disorders). Underlying mechanisms of these inequalities are to date not fully understood. This study aimed to investigate if (1) there are socioeconomic differences with regard to type of treatment and (2) if the socioeconomic status modifies the association between treatment and subsequent inpatient care or suicide attempt, respectively, in individuals with CMDs.

**Methods:**

The study population comprised 66,097 individuals aged 18–59 on sick-leave due to a CMD during 2006 in Sweden. Cox regression with a follow-up from 2007 through 2010 estimated crude and multivariate hazard ratios (HR) with 95% confidence intervals (CI).

**Results:**

Individuals with sickness absence due to CMDs and a higher educational level were had a lower proportions of specialised health care and combined psychiatric medication than their counterparts with lower education. However, if high educated CMD patients received more combined medication, associations with subsequent mental inpatient care (*p* < 0.01) and suicide attempt (*p* < 0.05) were stronger than for their counterparts with low education. Moreover, previous inpatient care due to mental disorders was associated with higher HRs of subsequent suicide attempt in CMD patients with high education (HR 5.88; CI 3.02–11.45) compared to those with low education (1.96; 1.06–3.60).

**Conclusion:**

Findings suggest that socioeconomic inequalities shape differences in treatment measures and mental health development in individuals with CMDs. These differences might signal discrepancies in treatment per se or reflect morbidity differences requiring different treatment regimens, or may be due to the fact that different diagnoses are given in different educational strata due to differential role of stigma.

## Introduction

Common mental disorders (CMDs), which are usually defined as depressive, anxiety, and stress-related disorders, are highly prevalent [1–3]. By 2020, unipolar depression e.g., is anticipated to be the main reason for lost disability adjusted life years in developed countries [4]. In many European countries, CMDs form one of the main reasons for sickness absence (SA) [5]. Individuals on sickness absence due to CMD have a high risk for adverse outcomes like permanent exit of the labour market including disability pension [5–7], or unemployment [8]. The risk of higher health care consumption due to CMDs is also increased [9]. This is especially pronounced for inpatient care [10], due to suicide attempts [11, 12]. Furthermore, CMDs are associated with higher mortality [7, 13], mainly due to suicide [11–13].

There seems to be a socioeconomic gradient with regard to both the occurrence of CMDs and sickness absence due to these disorders [14–18]. Moreover, socioeconomic status seems to contribute to inequalities in health care and treatment and in the development of mental illness in individuals with these disorders. A higher use of antidepressants and utilisation rate of primary health care have been reported in individuals with socio-economic disadvantages, even at given symptoms of depression and anxiety [19]. Additionally, newly marketed drugs seem to be prescribed more often to subjects with higher socio-economic status [20], while people with lower socio-economic status have been shown to be more often treated with drugs that are frequently associated with major side effects like sedation [21].

Underlying mechanisms of these inequalities are to date not fully understood. Potential explanations could indeed be socio-economic inequalities in adequate treatment, but also differences in the level of morbidity. Naturally, a number of other individual characteristics like sociodemographic factors and the type of diagnosis may influence these associations and have to be taken into consideration. Still, to the best of our knowledge no study to date has investigated if socioeconomic disadvantages shape inequalities in mental health development via differences in treatment in individuals with CMDs.

This study aimed to investigate if (1) there are socioeconomic differences with regard to type of treatment (six measures) and (2) if the socioeconomic status modifies the association between treatment and subsequent adverse mental health outcome in individuals with sickness absence due to common mental disorders (CMDs), while controlling for a number of sociodemographic and medical factors. Adverse mental health outcome was defined as inpatient care due to mental disorders or due to suicide attempt.

## Methods

### Study population

The study population of this prospective, population-based cohort study includes all 66,097 non-pensioned individuals alive, living in Sweden, and aged between 18 and 59 years on 31 December 2005 who had a new, incident sickness absence spell due to a CMD during 2006 (baseline). Individuals were followed with regard to inpatient health care due to mental disorders or suicide attempt from 1 January 2007 until 31 December 2010.

Data on individuals from 1 January 2000 (before inclusion) and up to 31 December 2010 were linked at an individual level, using the unique, de-identified personal identification number for all Swedish residents. The data were obtained from registers from the following three agencies:

1. Statistics Sweden: The Longitudinal Integration Database for Health Insurance and Labour Market Studies (LISA); this database contains sociodemographic information on age, sex, country of birth, education, region of residence, family situation and length of unemployment.
2. The Social Insurance Agency: The Micro-data for Analyses of Social Insurance (MiDAS) register provides data on the date, grade and diagnoses of sickness absence and disability pension.
3. The National Board of Health and Welfare: The National Patient Register (NPR) contains data on diagnosis and date of in- and specialized outpatient care from 1973 and from 2001, respectively; the Cause of Death Register (CDR) includes information on the date and cause of death from 1960; and the Prescribed Drug Register (PDR) holds data on the prescription of dispensed psychiatric medication (date of dispensing and type), from July 2005 and onward.

### Diagnostics

All diagnostic codes were defined according to the 10th Revision of the International Classification of Diseases, (ICD-10). The diagnoses used to define the study population (SA due to CMDs in 2006) were depressive disorders (F32: depressive episode and F33: recurrent depressive disorder), anxiety disorders (F40: phobic anxiety disorder, F41: other anxiety disorders, and F42: obsessive–compulsive disorder), and stress-related disorders (F43: reaction to severe stress and adjustment disorders). For the patient’s CMD diagnosis in the analysis, the diagnosis recorded for the first sickness absence spell due to a CMD in 2006 was used. Mental diagnoses comprised ICD 10 codes of F00-99 and somatic diagnoses were defined as any ICD 10 code other than mental. The Anatomical Therapeutic Chemical (ATC) Classification System codes N06A, N05B, and N05C were used for measuring prescriptions of antidepressants, anxiolytics, and hypnotics/sedatives, respectively.

### Outcome measures

Outcome measures are defined as future inpatient care due to mental disorders (ICD 10 codes F00-99) and suicide attempt (ICD10 codes X60-84).

### Covariates

The following socio-demographic variables were included as covariates: country of birth, age, sex, family situation, type of living area; all measured at the end of the year prior to baseline (i.e. 31 December 2005). Information on receipt of unemployment benefits in the year preceding baseline was used as a measure of labour market marginalization. Morbidity related covariates were measured as follows: type of CMD (depressive, anxiety, or stress-related) and net number of sickness absence days in 2005). Categorization of covariates is indicated in Table 1.

**Table 1:** Descriptive statistic of all 66,097 non-pensioned individuals with sickness absence (SA) due to common mental disorders (CMD) in Sweden in 2006

Characteristics	Percent
Socio-demographic characteristics	
Sex	
Female	69.2
Male	30.8
Age (years)	
18–24	6.7
25–34	25.8
35–44	32.1
45–54	25.0
55–59	10.4
Education (years)	
Low (≤9)	13.0
Medium (10–12)	51.9
High (>12)	35.1
Type of living area	
Big cities	38.5
Medium sized cities	35.1
Small cities/villages	26.4
Country of birth	
Sweden	86.3
Other Northern European countries	3.2
EU-25 without Northern Europe	2.1
Rest of the world	8.4
Family status^a^	
Married/cohabitating with children	40.8
Married/cohabitating without children	10.3
Single with children	14.5
Single without children	34.4
Prior labour market marginalisation	
Number of unemployment days in 2005	
0	82.7
1–90	8.2
>90	9.1
Morbidity related characteristics	
CMD diagnoses at first SA	
Depressive	36.9
Anxiety	11.8
Stress-related	51.2
Number of SA days in 2005	
0	73.8
1–90	21.1
>90	5.2

^a^Children living at home

### Exposure measures

Treatment was measured in five different exposure measures: previous in- and specialized outpatient care for mental and somatic diagnoses (none vs. any from 2000–2006 to 2001–2006, respectively; four variables), and type of psychiatric medication (one type only, more than one type, or none as indicated in Table 2). Socioeconomic status was measured as educational level according to the number of years of education (based on the educational system in Sweden): low (<9 years, compulsory education), medium (10–12 years, upper-secondary school) and high educational level (>12 years, university and higher education).

**Table 2:** Treatment characteristics of all 66,097 non-pensioned individuals with sickness absence (SA) due to common mental disorders (CMD) in Sweden in the year 2006, stratified by educational level

Treatment characteristics	All	Educational level^a^		
Specialised outpatient care diagnoses		Low	Medium	High
Somatic				
None	18.2	17.3a	18.3b	18.4b
Any	81.8	82.7a	81.7b	81.6b
Mental				
None	80.1	75.2a	79.5b	83.0c
Any	19.9	24.8a	20.5b	17.0c
Inpatient care diagnoses				
Somatic				
None	65.3	62.8a	64.8b	67.0c
Any	34.7	37.2a	35.2b	33.0c
Mental				
None	92.4	89.1a	92.0b	94.1c
Any	7.6	10.9a	8.0b	5.9c
Psychiatric medication				
Non	36.4	30.6a	35.9b	39.1c
Antidepressants only	18.8	18.6a, b	19.3b	18.2a
Anxiolytics only	3.3	4.0a	3.4b	2.9c
Hypnotics/sedatives only	8.0	8.2a	7.4b	8.9a
Combinations	33.5	38.5a	33.9b	30.9c

a, b, c: Similar letters for the various variable categories across columns denote no significant differences (*p* < 0.05). Differing letters denote significant differences

^a^Low (<9 years of education), medium (10–12 years) and high educational level (<12 years)

### Sickness insurance in Sweden

Swedish sickness insurance covers all people above the age of 16 who are living in Sweden and have at least a minimum annual income from work [22]. Compensation can be provided for individuals with reduced work capacity of at least 25% due to a disease or injury by either the employer or the Social Insurance Agency (SIA). Employees receive sick pay from day 2–14 of a sickness absence spell from the employer (the first day being a qualifying day). From day 15 employees get sickness benefit from SIA. From the eighth day of a sickness absence spell onwards, a certificate from a physician is required. Unemployed individuals and individuals on parental leave can be granted sickness benefit from SIA from the second day of a sickness absence spell, whereas self-employed individuals receive sick pay from SIA according to which insurance coverage they had chosen. For this study, information on sickness absence from SIA has been used.

### Statistical methods

Cross tabs were calculated and the Pearson’s Chi square test and *z* test performed to assess possible differences in treatment characteristics by level of education. Risk estimates of future inpatient care due to mental disorders or suicide attempt were assessed by using Cox proportional hazard regression models after assuring that the proportional hazard assumption was fulfilled. Time to inpatient care was defined as the time between the start of follow-up (1 January 2007) and when inpatient care due to mental disorders or suicide attempt occurred, provided that this occurred before the end of follow-up (31 December 2010). Censoring was due to emigration, death, or end of follow-up, whichever came first. Analyses were adjusted for sociodemographic variables, the specific CMD diagnosis and the number of sickness absence days. Potential interactions between level of education and treatment characteristics were tested using the partial likelihood ratio test. Analyses were conducted using IBM SPSS Statistics version 22.0.

### Ethical considerations

The project was approved by the regional ethical review board in Stockholm (approval number: 2007/762-31).

## Results

Two-thirds of subjects on sickness absence due to a CMD in 2006 were women. Most of the individuals in the study population were between 35 and 54 years of age, with secondary education as highest registered educational level, lived in big cities, were born in Sweden, and were married or cohabitating with children living at home (Table 1). Most of the subjects on sickness absence due to a CMD did not have any unemployment days or SA days in 2005, the year prior to baseline. The most common category of CMD in the study population was “stress-related” (Table 1).

The majority had previous specialised outpatient care due to somatic diagnoses in the years preceding and including baseline, but no previous specialised outpatient care due to mental reasons or inpatient care due to any reason in the years before (Table 2). The most frequent kind of prescription for subjects on SA due do a CMD was a combination of at least two types of psychiatric medications, i.e. antidepressants, anxiolytics or sedative/hypnotics (Table 2).

Stratification by educational level revealed that individuals with low educational level had higher proportions of treatment in terms of specialised health care than their counterparts with high education (Table 2). Moreover, medication with anxiolytics only and combined psychiatric medication was more common among individuals with low educational level (Table 2).

Stress-related SA diagnoses were more common in individuals with higher education, and depressive and anxiety disorders were more frequently diagnosed in patients with lower education. A depression-related diagnosis was given to 40.5, 37.9 and 34.2% of subjects with low, medium and high education, respectively. The corresponding proportions were 15.1, 13.2, and 8.7% for anxiety, and 44.4, 48.9, and 57.1%, for stress-related diagnoses. These differences were significantly different (*p* < 0.001).

During the 4 years of follow-up (2007–2010), 1.5% of individuals with sickness absence due to CMD had an inpatient care due to mental disorders, and 0.4% due to suicide attempt. In the multivariate analyses, specialised outpatient care due to mental disorders, inpatient care regardless of diagnosis, and having been prescribed combined medication were associated with a higher risk of future inpatient care due to mental disorders (Table 3) and due to suicide attempt (Table 4), compared to having had no such care/medication. There was a significant interaction of education with the applied medication regime in terms of subsequent inpatient care due to mental disorders (*p* = 0.007), and in terms of subsequent suicide attempts (*p* = 0.026).

**Table 3:** Hazard ratios (HR) and 95% confidence intervals (CI) for subsequent inpatient care due to mental disorders in the years 2007–2010, related to treatment characteristic in all 66,097 non-pensioned individuals with sickness absence (SA) due to common mental disorders (CMD) in Sweden in 2006, stratified by educational level

Treatment characteristics	Low education		Medium education		High education		*p*_interaction_**
Specialised health care diagnoses	HR	95% CI	HR	95% CI	HR	95% CI	
Outpatient care (somatic)							
None	1		1		1		0.30
Any	1.59	0.85–3.00	0.99	0.77–1.29	0.97	0.65–1.46	
Outpatient care (mental)							
None	1		1		1		0.65
Any	1.48	1.04–2.11	1.89	1.56–2.29	1.76	1.29–2.41	
Inpatient care (somatic)							
None	1		1		1		0.52
Any	1.66	1.20–2.31	1.39	1.17–1.65	1.34	1.02–1.75	
Inpatient care (mental)							
None	1		1		1		0.50
Any	3.71	2.61–5.27	3.31	2.73–4.03	3.86	2.80–5.31	
Psychiatric medication							
None	1		1		1		0.007
Antidepressants only	1.58	0.87–2.89	1.73	1.28–2.34	2.19	1.31–3.66	
Anxiolytics only	1.46	0.54–3.92	0.45	0.17–1.23	4.22	2.02–8.80	
Hypnotics/sedatives only	1.32	0.57–3.02	1.49	0.96–2.30	0.81	0.31–2.12	
Combination	2.27	1.35–3.82	2.47	1.90–3.22	3.81	2.42–6.00	

Mutually adjusted for all health care characteristics and additionally adjusted for sex, age, type of living area, country of birth, family status, number of unemployment days 2005, category of common mental disorder at first sickness absence 2006, and number of sickness absence days in 2005

** *p*_interaction_ resembles the *p* value for the interaction between specific treatment options and socioeconomic status with regard to the specific outcome measure

**Table 4:** Hazard ratios (HR) and 95% confidence intervals (CI) for subsequent inpatient care due to suicide attempt in the years 2007–2010, related to treatment characteristic in all 66,097 non-pensioned individuals with sickness absence (SA) due to common mental disorders (CMD) in Sweden in 2006, stratified by educational level

Treatment characteristics	Primary education		Secondary education		Tertiary education		*p*_interaction_**
Specialised health care diagnoses	HR	95% CI	HR	95% CI	HR	95% CI	
Outpatient care (somatic)							
None	1		1		1		0.011
Any	–	–	1.06	0.62–1.78	0.80	0.33–1.95	
Outpatient care (mental)							
None	1		1		1		0.090
Any	2.05	1.11–3.78	1.72	1.20–2.46	2.73	1.34–5.54	
Inpatient care (somatic)							
None	1		1		1		0.300
Any	2.59	1.42–4.71	1.71	1.24–2.36	1.31	0.74–2.33	
Inpatient care (mental)							
None	1		1		1		0.019
Any	1.96	1.06–3.60	4.12	2.87–5.91	5.88	3.02–11.45	
Psychiatric medication							
None	1		1		1		0.026
Antidepressants only	1.80	0.69–4.70	1.20	0.68–2.12	1.53	0.40–5.92	
Anxiolytics only	–	–	0.34	0.05–2.52	7.98	1.76–36.09	
Hypnotics/sedatives only	–	–	0.88	0.34–2.30	0.98	0.11–8.78	
Combination	1.98	0.84–4.64	2.25	1.41–3.58	4.25	1.40–12.90	

Mutually adjusted for all treatment characteristics and additionally adjusted for sex, age, type of living area, country of birth, family status, number of unemployment days 2005, category of common mental disorder at first sickness absence 2006, and number of sickness absence days in 2005

** *p*_interaction_ resembles the *p* value for the interaction between specific treatment options and socioeconomic status with regard to the specific outcome measure

In analyses stratified by educational level, treatment with antidepressants only, anxiolytics only, or a combination therapy, the risk of future inpatient care due to mental disorders was higher compared to those without medication. The higher the educational level, the more pronounced was the association between type of medication and future inpatient care due to mental disorders (Table 3). Associations between combined medication and subsequent suicide attempt were also significant (*p* < 0.01). These associations were stronger in case of higher educational level. Additionally, inpatient care due to mental disorders was related to a higher risk of future suicide attempt and risk estimates associated with higher education were higher than risk estimates related to lower education (Table 4).

## Discussion

Individuals with sickness absence due to CMDs and a high educational level had lower proportions of specialised health care and combined psychiatric medication than their counterparts with low education. However, if high educated CMD patients received combined medication, risk estimates for subsequent inpatient care due to mental disorders or suicide attempts were higher compared to estimates for patients with lower education.

The modifying effect of socioeconomic status on the association between treatment and subsequent inpatient care due to mental disorders or suicide can be interpreted in two different ways: (1) morbidity-related, or (2) treatment-related. Applying the first way of interpretation (morbidity-related), CMD patients with lower education received different diagnoses (i.e. more often anxiety and depressive disorders in case of lower education, and more often stress related diagnoses in case of higher educated individuals (data not shown). Previous studies suggest a worse prognosis in terms of mortality in individuals with stress-related diagnoses in comparison to individuals with depressive disorders [13]. Moreover, lower educated individuals received more often any medication or combined medication or specialised health care than individuals with higher education. These differences might reflect a higher severity of the underlying disorder in lower educated patients. Higher risk for CMD and especially more severe CMDs in subjects with lower socio-economic status has been previously reported [14, 16, 17].

If, however, higher educated subjects were prescribed combined medication, they had a worse mental health development than individuals with similar treatment regimens but lower education. Moreover, the risk for suicide attempt in case of a former inpatient care due to mental disorders was higher in higher educated versus lower educated patients. These findings suggest that if higher educated patients had a more severe form of CMD and more specialised health care and combined psychiatric medication, they were more prone to adverse outcomes. In other words, using the “morbidity” explanatory model, socioeconomic status seems to modify the effect of morbidity on subsequent adverse health outcome.

The alternative (diagnosis- and treatment-related) explanation is based on the hypothesis, that diagnoses and treatment are given differently according to the educational level, at a given burden of disease. It is likely that a stress-related diagnosis is given more often to patients with higher education due to its better “image” and the lower risk for stigmatization, which is usually attached to anxiety and depressive disorders [23, 24]. Moreover, more stigmatising attitudes seem to be related to psychiatric medication than to e.g. psychotherapy [25]. This could be the reason why psychiatric medication and a combination of psychiatric medication were less often prescribed for patients with higher education. Higher educated patients are also known to be more often involved in making shared decisions regarding their treatment [26]. It is therefore likely that either treatment with psychiatric medication is not as frequently offered to them, or they are more likely to decline taking medication. Other studies have found less drug treatment in patients with CMD with higher socioeconomic status, even at given symptoms for anxiety or depression [19]. It is therefore likely, that receiving an adequate diagnosis and treatment is more often delayed in CMD patients with higher education. This delay and the further developed disease would lead to the worse outcome when having more specialised health care/combined psychiatric medication. Using this explanation, the effect of diagnosis and treatment regarding subsequent adverse mental health outcome is modified by socioeconomic status.

### Strengths and limitations

A strength of the study is the prospective and population-based cohort design, the large cohort of more than 66,000 subjects with sickness absence due to CMD, the detailed data on past and future health care, and the long follow up (4 years), and no loss to follow-up. The quality of the Swedish administrative registers, including the sickness absence register [27] is generally high [28, 29]. Additionally, diagnoses related to the exposure have been set by physicians, that is, they were not self-reported. Exposure, confounders, and outcome measures were recorded independently from each other. Moreover, the size of the study population offered satisfactory statistical power for the analyses, including a wide range of possible confounders.

Limitations include that the information on in- and specialised outpatient care mainly covers morbidity of more pronounced medical severity. Information on individuals treated within general practitioners in primary health care is therefore only available through information on sickness absence and prescribed medication. Furthermore, we did control for but did not determine risk estimates for the different CMDs including a variety of different disorders like depressive, anxiety, and other stress-related disorders. This decision was based on the fact that such analyses would have been considerably underpowered. We have used education as an indicator (proxy) of the construct of socioeconomic status. Education is one example of such a proxy. Other examples are occupational status and income. As with all proxies, there is a vivid discussion about the best choice of an indicator of SES. We chose education as it is often described as a reliable indicator of SES due to the fact that it is stable, established in early adulthood and not affected by chronic diseases [30]. Still, it should be noted that—regardless of the choice of an indicator—there is a risk that a single measure might not reflect all dimensions of one’s socioeconomic status. The study included information on individuals on sickness absence due to CMDs with benefits from the Social Insurance Agency. This implies that employees contributed with information on their sickness absence after 14 days, as the first 2 weeks are covered by the employer. As most sickness absence spells due to CMDs last much longer than 2 weeks [31], the loss of information might be negligible.

## Conclusions

Findings suggest that socioeconomic inequalities shape differences in treatment measures and mental health development in individuals with sickness absence due to CMDs. These differences might signal discrepancies in treatment per se or reflect morbidity differences requiring different treatment regimens.
